# The Role of The Tumor Microbiome in Tumor Development and Its Treatment

**DOI:** 10.3389/fimmu.2022.935846

**Published:** 2022-07-15

**Authors:** Yan Chen, Fa-Hong Wu, Peng-Qiang Wu, Hong-Yun Xing, Tao Ma

**Affiliations:** ^1^ Department of Hematology, The Affiliated Hospital of Southwest Medical University, Luzhou, China; ^2^ Department of General Surgery, Hepatic-Biliary-Pancreatic Institute, Lanzhou University Second Hospital, Lanzhou, China

**Keywords:** tumor microbiome, clinical features, immunity, treatment, biomarker, GEN-001

## Abstract

Commensal bacteria and other microorganisms that reside in the human body are closely associated with the development and treatment of cancers. Recently, tumor microbiome (TM) has been identified in a variety of cancers such as pancreatic, lung, and breast cancers. TM has different compositions in different tumors and has different effects on tumors. TM plays an important role in the formation of the tumor microenvironment, regulation of local immunity, and modification of tumor cell biology, and directly affects the efficacy of drug treatment for tumors. TM is expected to be a biomarker for tumors, and engineered tumor-targeting bacteria and anti-cancer microbial agents (GEN-001) have an important role in the treatment of tumors. This paper reviews the relevant studies on TM in recent years and describes its distribution in different tumors, its correlation with clinical features, its effect on local immunity, and the research directions of TM in tumor treatment.

## 1 Introduction

The collective genomes and by-products of all microorganisms that inhabit the human body are called the human microbiome, which includes bacteria, viruses, fungi, etc (1). Located in the human mouth, skin, gut, and other parts of the body, these microbiota affect how you digest food, help train your immune system, and may even influence your mood and behavior (2–4). There is an evolutionary partnership between humans and the microbiota that is essential for metabolism, tissue development, and host defense (5). Microbiota is directly or indirectly associated with metabolic disorders, cardiovascular diseases, neurological disorders, and even psychological disorders such as schizophrenia (6–8). In recent years, more attention has been paid to its role in cancer.

Microbiota plays an important role in the development, diagnosis, and treatment of many human tumors (9). Many microorganisms are responsible for the development of cancer in humans (10). *Helicobacter pylori* (HP) infection can lead to gastric inflammation and even gastric malignancies such as gastric ulcers, gastric cancer (GC), and gastric mucosa-associated lymphoid tissue (MALT) lymphoma (11). Microbiota, especially gut microbiota, modulates response to cancer treatment and susceptibility to toxic side effects (12). The gut microbiota can affect local and distant tumors by influencing the immune environment, inflammation, and metabolic patterns of the tumor9. Gut microbiota has also been associated with the development and treatment of acute leukemia and may predict the development of graft-versus-host disease (GVHD) in allogeneic hematopoietic stem cell transplant patients (13). As more studies of the microbiota were conducted, it was discovered that the microbiota was also present within tumor tissues once thought to be sterile (14). This paper focuses on an overview of the current status of research on the tumor microbiome (TM) and the opportunities and challenges it faces.

## 2 Microbiomes are everywhere, even inside tumor cells

The microbiota in the human body is surprisingly large. An adult man weighing 70 kg consists of about 3.0x10^13^ human cells, and the number of microorganisms symbiotic with this person is about 3.8x10^13^ - the same order of magnitude as the number of human cells (15). The presence of bacteria in tumor tissues was identified more than 100 years ago (16). In recent years, microbiota has been found in a variety of cancer tissues, including breast, lung, colorectal, and prostate cancers (PCA) (17–20). However, characterization of tumor microorganisms remains challenging due to their extremely low biomass (16). In recent years, with the application of next-generation sequencing technology, the characteristics of the internal microbiota of tumors have been studied more intensively.

To determine the presence or absence of microbiome within the tumor tissue, the first step is to rule out the possibility of contamination after the sample leaves the organism; after all, microorganisms are everywhere. There are two main types of contaminants when performing sequencing tests (21). External contamination comes from outside the sample being tested, including the researcher’s body, laboratory surfaces, air, instruments, and reagents (21). Cross-contamination of samples may occur during sample processing or sequencing. Davis et al. introduced and validated an easy-to-use open source R package, decontam, which identifies and removes external contaminants from sequencing data (21). It has been found that a unique microbiome exists in the placenta and that the placental microbiome profile is most similar to that of the human oral microbiome. Women with severe periodontal disease are also at higher risk for adverse pregnancies, and periodontal pathogens may colonize the placenta through hematogenous infection (22–25). Davis et al. applied decontam to a recently published dataset to confirm and extend their conclusion that there is little evidence of an indigenous placental microbiome and that some low frequency taxa that appear to be linked to preterm birth are contaminants (21, 26). Negative controls processed with the samples and paraffin blocks without tissue (taken from the edges of the paraffin blocks used in the study) also need to be tested to exclude contamination (16). Nejman et al. studied 1526 tumors of seven cancer types and their adjacent normal tissues and found that each tumor type has a unique microbiome composition, with breast cancer (BC) having a particularly rich and diverse microbiome16. Most of the bacteria within the tumors were intracellular and were present in both cancer cells and immune cells (16). Interestingly, electron microscopy showed that the bacteria within the cells were largely devoid of cell walls, suggesting an L-form–like state (16, 27, 28). Mycoplasma is also a member of the TM and also has no cell wall. It has been shown that in hepatocellular carcinoma (HCC), mycoplasma infection promotes tumor progression through the interaction of the mycoplasma protein p37 with epithelial cell adhesion molecules (29). In PCA, mycoplasma has also been shown to promote its progression, as we will discuss in detail in section 3.1 (30).

**Figure 1:**
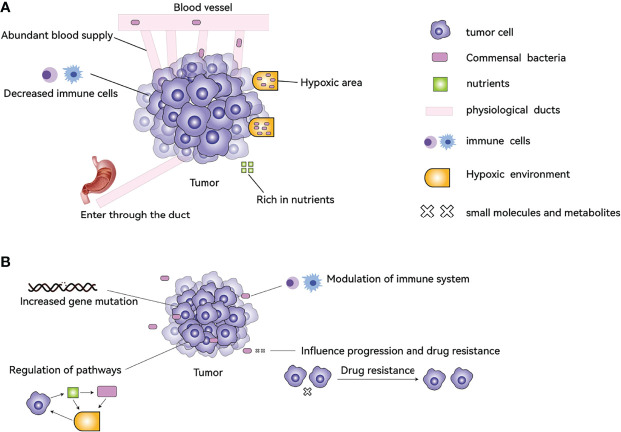
The causes of TM formation in tumors and its effects on tumors. **(A)** The abundant blood supply, hypoxic environment, abundant nutrients, and reduced immune cells help the bacteria to colonize the tumor tissue. Bacteria can also enter through the ducts, for example, from the duodenum into the pancreas. **(B)** TM can affect tumor characteristics by increasing gene mutations, regulating the function of immune cells, modulating signaling pathways, and influencing drug resistance.

It is certain that some types of cancer cells contain microbiota, but where do they come from? After tumor formation, the special tumor microenvironment attracts the microbiota to accumulate? Or is some microbiota itself involved in the process of tumor formation? Both of these possibilities exist. Why do bacteria gather in tumor tissues and cells after tumor formation in the human body? There are several reasons: 1. Cancer cells evade the recognition of immune cells through various mechanisms, resulting in insufficient strength of immune cells inside the tumor, and the interior of the tumor provides a refuge for microorganisms to avoid immune clearance. 2 The hypoxic nature of the interior of many solid tumors results in a low oxygen content compared to normal tissue, providing an environment for anaerobic bacteria to survive. 3. Highly disorganized neovascularization, slow blood flow, and blood leakage inside the tumor lead to bacteria in the blood circulation entering the tumor tissue. 4. Bacteria enter directly through ducts that are connected to the outside world, for example, bacteria enter the pancreas from the duodenum. 5. The tumor tissue is highly nutritious inside and has some metabolites (such as ribose, aspartic acid, etc.) to attract bacteria (**Figure 1A**) (31, 32).

In a study of pancreatic cancer (PC), it was found that the gut microbiota can colonize pancreatic tumors, altering tumor bacterial composition and modulating immune function, ultimately affecting the natural course and survival of PC (32). Microorganisms are involved in tumor formation, and 10 microorganisms were identified by the International Agency for Research on Cancer as carcinogenic to humans in 2012. They are *Schistosoma haematobium*, HP, *Opisthorchis viverrini, Clonorchis sinensis*, human papillomavirus (HPV), hepatitis B virus (HBV), hepatitis C virus (HCV), human T-cell lymphotropic virus type 1 (HTLV-1), human herpes virus type 8 (HHV-8; also known as Kaposi’s sarcoma herpes virus), and Epstein-Barr virus (EBV) (10, 33). In 2020, human immunodeficiency virus-1 was added to the list34. In 2018, about 2.2 million new cancer cases were attributed to infections of microorganisms, accounting for 13% of all cancer cases, mainly HP infection leading to GC, HPV interference leading to cervical cancer, and HBV and HCV infection leading to HCC (34). HP produces multiple virulence factors such as cytotoxin-associated gene A (CagA) and its pathogenicity island (Cag PAI), and vacuolating cytotoxin A (VacA), which may dysregulate intracellular signaling pathways in the host and decrease the threshold of tumor transformation11. Péneau et al. analyzed the genome of 177 tumor tissues from HCC patients and found that HBV gene integration was present in 88% of patients, with the vast majority (82%) of tumors having at least one clonal HBV integration, compared to 11% in non-tumor liver tissue (35). A high percentage of HBV integrations in tumors affect cancer driver genes, such as TERT, TP53, and MYC, and tumors with high numbers of HBV integrations are associated with poor prognosis (35). HPV expresses oncogenic proteins E6 and E7, and gene expression levels of oncogenes and pathways are higher in HPV^+^ cervical cancer tissues compared to cervical cancer tissues without HPV integration (33, 36).

The link between TM and cancer has been demonstrated by four main mechanisms: 1. increased gene mutations directly promoting tumorigenesis, 2. regulation of oncogenes or oncogenic pathways, 3. modulation of the host immune system, and 4. production of small molecules or metabolites that influence cancer development, progression, and response to therapeutic agents (**Figure 1B**) (32, 33, 35–38).

## 3 Studies of TM in different cancers: distribution of microbiota, relationship with clinical features, effects on tumor immunity

### 3.1 Prostate Cancer

Many studies have confirmed the presence of microbiota in PCA tissues that are different from normal prostate tissues and that some mycoplasma and viruses can promote the development of PCA. In a study of PCA, researchers tested 170 prostate tissue core samples from 30 cancer patients for 16S rDNA gene sequences and found the presence of 83 different microorganisms, but because most individual tissue core samples were negative, the researchers concluded that there was regional heterogeneity of bacteria and a lack of a universal or ubiquitous prostate flora (20). After further culturing tissue samples from the patients, they found that the species present in the prostate had a “non-culturable” nature, and the researchers concluded that the 16S rDNA sequencing results may have come from non-viable bacteria (20). There were significant differences in specific microbial populations in tumor/peri-tumor and non-tumor prostate specimens, with *Propionibacterium* spp. being the most abundant among the genera and *Staphylococcus* spp. being more abundant in the tumor/peri-tumor tissues (39). Feng et al. studied the macrogenome and supertranscriptome of 65 PCA tumors and benign paracancerous tissues, and found that *Escherichia, Propionibacterium, Acinetobacter*, and *Pseudomonas* were abundant, and the expression profile of 10 Pseudomonas genes was correlated with those of eight host small RNA genes; three of these RNA genes may be negatively correlated with metastasis, and *Pseudomonas* infection may impede metastasis (40). However, further studies are needed. Some mycoplasma infections may promote the development of PCA. *Mycoplasma genitalium* and *Mycoplasma hyorhinis* infections can lead to increased migration and invasion of human benign prostate cells and malignant transformation (30). Testing of specimens from patients with PCA and benign prostate disease revealed significant differences in the prevalence of *Mycoplasma genitalium* infection between the PCA cohort and the benign prostatic hyperplasia cohort (41). Infection with viruses such as HPV and *polyomavirus BK* may also promote the occurrence of PCA (42, 43).

Some microorganisms can be used as biological markers and therapeutic targets for PCA, and the TM of PCA can influence the efficacy of its immunotherapy. Human endogenous retroviruses (HERV) can be used as potential biomarkers and therapeutic targets for prostate, breast, and colon cancers (44). HERV-K Gag expression was significantly increased in malignant regions of men with PCA compared to benign regions and men without PCA, and 85.2% of PCA donors showed upregulation of HERV-K Gag RNA associated with malignancy (45). A patient-derived prostate-specific uropathogenic *Escherichia coli* named CP1, when combined with anti-PD-1 immunotherapy, increases survival and reduces tumor burden in MYC and PTEN-mutated PCA models (46). CP1 also increases T-cell toxicity and immune death of tumor cells, and CP1 increases infiltration of activated CD8 T cells, Th17 T cells, mature dendritic cells, M1 macrophages, and NK cells into tumors, and intraurethral administration of CP1 specifically enters and colonizes the tumor without causing any systemic toxicity (46). There have also been many studies using microbes as targets for PCA (47–49).

### 3.2 Pancreatic Cancer

The presence of microbiota similar to that of duodenum in PC tissue can affect the outcome and prognosis of PC. A study of 113 human PC samples and 20 normal human pancreas samples found that bacterial DNA was detected in 86/113 (76%) PC samples and in 3/20 (15%) normal pancreas controls (38). The most common microbial species (51.7%) belonged to the class *Gammaproteobacteria*; most were members of the *Enterobacteriaceae* and *Pseudomonadaceae* families, and patients who underwent pancreatic duct instrumentation had significantly more bacteria in their tumors than those who did not undergo instrumentation. *Proteobacteria* are abundant in the duodenum, and retrograde bacterial migration from the duodenum to the pancreas may be the source of bacteria within the tumor tissue (38). A study by Pushalkar et al. also found that fluorescently labeled *Enterococcus faecalis*, gfp-labeled *Escherichia coli* can translocate from the intestine to the pancreas (50). They analyzed PC tissues and found that *Proteobacteria* (45%), *Bacteroidetes* (31%), and *Firmicutes* (22%) were the most abundant and prevalent in all samples, and that the bacterial composition in human PC was different from that of the normal human pancreas (50). In a mouse model, aseptic colonization reduced pancreatic dysplasia, intratumoral fibrosis, and pancreatic weight in mice compared to control mice. Tumor burden was reduced by approximately 50% in mice treated with an ablative oral antibiotic regimen, and bacteria promoted progression of pancreatic tumorigenesis in both pre-infiltrative and infiltrative models (50). The microbiome promoted the progression of PC by inducing peritumoral immunosuppression, and microbial ablation led to a significant increase in the proportion of intra-tumoral T cells and a decrease in the proportion of myeloid-derived suppressor cells (MDSC). Both CD4^+^ and CD8^+^ T cells in the tumors of antibiotic-inactivated mice also showed increased expression of PD-1 and CD44, and the use of feces from PC mice repopulated with microbiome after antibiotic ablation reversed the immunogenic changes in the tumors associated with bacterial ablation. Whole-pancreatic Nanostring arrays confirmed that genes associated with T-cell proliferation and immune activation were upregulated in tumors from antibiotic-treated mice (50).

Most patients with pancreatic adenocarcinoma (PDAC) survive for less than 5 years, but some patients survive for a long time (51). A study compared the TM of long-term survivors (LTS, median survival of 10.1 years) who survived more than 5 years after surgery with short-term survivors (STS, median survival of 1.6 years) who survived less than 5 years after surgery (32). The alpha-diversity of the TM was found to be significantly higher in LTS patients compared to STS, and overall survival was significantly longer in patients with high alpha-diversity (median survival: 9.66 years) than in patients with low alpha-diversity (median survival: 1.66 years). Clinico-pathological features, body mass index, gender, smoking, adjuvant therapy, and antibiotic use were not significantly associated with TM diversity (32).

LTS tumors were dominated by *Alphaproteobacteria Sphingobacteria* and *Flavobacteria*. In contrast, PDAC STS cases were predominant with *Clostridia* and *Bacteroidea*. The prognosis of PDAC patients with a higher abundance of the three genera, *Saccharopolyspora, Pseudoxanthomonas*, and *Streptomyces*, was significantly better. The diversity of the TM and the presence of these three genera in tumors may contribute to the antitumor immune response by favoring the recruitment and activation of CD8^+^ T cells. Compared with STS patients, LTS patients had greater densities of CD3^+^ and CD8^+^ T cells, and LTS patients had significantly higher numbers of granzyme B^+^ cells, while no significant differences were found in regulatory T cells, macrophages, or MDSC (32).

Pushalkar et al. showed that the gut microbiome can promote the progression of PC by inducing peritumor immunosuppression and that these gut microbiomes may translocate into the pancreas (50). Riquelme et al., on the other hand, found that some microorganisms in PC tissues (e.g., *Alphaproteobacteria, Sphingobacteria*, and *Flavobacteria*) may contribute to the anti-tumor immune response by favoring the recruitment and activation of CD8^+^ T cells, leading to a good prognosis (32). The TM is clearly associated with the development and progression of PC, and the use of anti-cancer therapy targeting these microorganisms is an important direction for future research.

### 3.3 Breast Cancer

Microbiota can be found in normal human breast tissue, breast cancer tissue, and benign breast disease tissue (52). The microbiota within BC is different from that of normal breast tissues. The bacteria *Methylobacterium radiotolerans* was relatively enriched in BC tissues, whereas the bacterium *Sphingomonas yanoikuyae* was relatively enriched in paired normal tissues, and the relative abundance of these two bacteria was inversely correlated in paired normal breast tissues, but not in BC tissues17. Another study found a higher relative abundance of Bacillus and *Enterobacteriaceae* in BC tissues (53). The proportion of *Pseudomonadaceae* and *Enterobacteriaceae* was much higher in BC tissues compared to other tissues. In contrast, *Propionibacterium* and *Staphylococcus* were the major components of healthy controls and tumor adjacent normal tissues, but were rare in BC tissues (54). BC has a richer and more diverse microbiota than other tumors such as PC and melanoma, with an average of 16.4 bacteria species detected per sample in BC, compared to an average of <9 detected in all other tumor types (16). The bacterial load and abundance in BC was higher than in normal breast samples, while normal breast tissue adjacent to the tumor was in between. The researchers further collected fresh breast tumor samples from five women who underwent breast surgery, cultured them, and grew 37 different bacterial species, 11 of which were consistent with the previous test results (16). Four freshly excised human breast tumor sections were cultured *in vitro* in the presence of fluorescently labeled d-alanine or dimethyl sulfoxide controls. D-alanine is an important component of the bacterial cell wall and is not utilized by mammalian cells, and intracellular labeling was detected in all four tumors, further supporting the hypothesis that viable bacteria are present in the tumors (16).

Significant differences in the TM were found between subtypes of BC, including Human epidermal growth factor 2 (HER2)^-^ vs. HER2^+^, estrogen receptor (ER)^-^ vs. ER^+^, and triple negative (TNG) vs. not-TNG (16). Compared with ER- BC, the abundance of seven genera (*Alkanindiges, Micrococcus, Caulobacter, Proteus, Brevibacillus, Kocuria, and Parasediminibacterium*) of ER^+^ BC was lower. The abundance of seven genera (*Clostridium, PRD01a011B, Alloprevotella, Stakelama, Filibacter, Blastomonas, Anaerostipes*) of HER2^+^ tumors was significantly higher than that of HER2- tumors. Six of the seven genera (except Micrococcus) that were relatively reduced in ER^+^ tumors were enriched in TNG BC (54). *Lymphovascular* invasion in BC was positively correlated with Lactobacillus and negatively correlated with *Alkanindiges*, whereas node-positive status was positively correlated with *Acinetobacter* and *Bacteroides* and negatively correlated with *Achromobacter* (54). A significant amount of TM was also found to be present in the BC model in mice, and removal of the TM obviously reduced lung metastasis but did not affect the growth of the primary tumor (55). Intratumoral bacteria (mainly *Staphylococcus* and *Lactobacillus*) carried by circulating tumor cells can promote BC cell lung metastasis by reorganizing the actin cytoskeleton to enhance resistance to fluid shear stress (55).

The microbiome in BC may affect the immune microenvironment within the tumor. Compared to normal controls, tumor tissues were enriched in total T cells, CD8^+^ T cells, natural killer (NK) cells, and neutrophils, but reduced in dendritic cells and macrophages (54). Three genera (*Methylibium, Pelomonas, Propionibacterium*) were identified as nodes in the microbiome-immune gene and microbiome-cytokine networks (54). In BC tissues, *Methylibium* showed a significant negative correlation with T cell abundance, the oncogene TRAF4 was negatively correlated with Staphylococcus, while the pro-angiogenic factor VEGF-A was positively correlated with *Pelomonas* and negatively correlated with *Bradyrhizobium* (54). *Fusobacterium nucleatum* (*F. nucleatum*) binds to BC samples *via* lectin Fap2-dependent binding, and inoculation with *F. nucleatum* inhibited the accumulation of tumor-infiltrating T cells and promotes tumor growth and metastatic progression (56). *Metronidazole* prevented tumor enlargement and pro-metastatic effects in mice inoculated with *F. nucleatum*56. The use of the intra-TM as a biomarker for assessing prognosis and for the treatment of BC is a future research direction.

### 3.4 Lung Cancer

The bacterial composition of the pulmonary microbiota is different from the gut or skin microbiota, but has considerable similarity to the upper respiratory tract and oral microbiota (57). People with lower microbial diversity have a higher risk of LC compared to those with higher oral microbiota diversity (58). Saliva of squamous cell carcinoma (SCC) and adenocarcinoma (AC) patients had significantly altered levels of *Capnocytophaga, Selenomonas, Veillonella*, and *Neisseria* compared to healthy controls, with significantly higher levels of *Capnocytophaga* and *Veillonella*, and elevated levels of these two bacteria in saliva of lung cancer (LC) patients could be potential biomarkers for disease detection (59). Microbiota in bronchoalveolar fluid also showed differences between patients with LC and those with benign mass-like lesions, with *Veillonella* and *Megasphaera genera* being significantly increased in patients with LC, showing potential as biomarkers for predicting LC (60). Normal lung tissue had lower microbiome alpha diversity than tumor tissue and non-tumor adjacent tissues, and a separate set of taxa were identified in SCC, in which *Acidovorax* was enriched in smokers (18). *Acidovorax* showed a higher abundance in SCC cases with TP53 mutations. This may be because tumors carrying TP53 mutations can impair epithelial function (18). A study compared the bacterial functions found in non-small cell lung cancers (NSCLCs) from 100 current smokers with those found in NSCLCs from 43 never-smokers. Seventeen pathways were found to be significantly enriched in the tumors of current smokers, pathways that degrade chemicals in cigarette smoke, and eight pathways related to biosynthesis of metabolites that can be used by plants, possibly because some plant-associated bacteria or their DNA are present in cigarette tobacco and therefore enriched in the lung tumors of smokers. Bacteria expressing these functions are mainly found in the *Proteobacteria, Actinobacteria*, and *Cyanobacteria phyla* (16).

The microbiota of the lung is not only distributed differently in LC and normal lung tissues, but also has a close association with the occurrence, prognosis, and immune modulation of LC. A study that sequenced lung tumor and normal samples from the same lobe/segment of 19 NSCLC patients found that tumor tissue had lower bacterial abundance and diversity than paired normal tissue (61). In normal tissues, a higher abundance of family *Koribacteraceae* was associated with increased recurrence-free (RFS) and disease-free survival (DFS), whereas higher abundance of families *Bacteroidaceae, Lachnospiraceae*, and *Ruminococcaceae* were associated with decreased RFS or DFS. However, the diversity and overall composition of tumor tissues were not associated with RFS or DFS61. This study suggested that the diversity and composition of the lung microbiota were not associated with LC prognosis, but the sample size was too small and needs to be expanded for further exploration.

Other studies suggest that the lung microbiome can influence the prognosis of LC. The lower airways of LC patients are enriched with oral taxa (*Streptococcus* and *Veillonella*) and the enrichment of these bacteria may be associated with upregulation of ERK and PI3K signaling pathways, and *in vitro* exposure of airway epithelial cells to *Veillonella, Prevotella*, and *Streptococcus* also led to upregulation of these signaling pathways (62). This lower airway dysregulation feature is more prevalent in the stage IIIB-IV tumor node metastasis LC group and is associated with poor prognosis. This dysregulation of the lower airway microbiota was associated with upregulation of the IL-17, PI3K, MAPK, and ERK pathways in the airway transcriptome, with *Veillonella parvula* being the most abundant taxon driving this association (63). *In vitro* experiments found that increased *Veillonella parvula* in the lower airway microbiota can lead to decreased survival, increased tumor burden, IL-17 inflammatory phenotype, and activation of checkpoint inhibitor markers (63). And the dysregulation of lung microbiota induced by *Veillonella parvula* led to the recruitment of Th17 cells, increased levels of IL-17 production, increased expression of PD-1^+^ T cells, and recruitment of neutrophils (63). Alterations in the LC microbiome modulate host immunity in LC and affect tumor progression and prognosis. Commensal bacteria from LC stimulated Myd88-dependent IL-1β and IL-23 production by myeloid cells, inducing proliferation and activation of Vγ6^+^Vδ1^+^γδ T cells, which produce IL-17 and other effector molecules to promote inflammation and LC cell proliferation (64).

Smoking and some genes (e.g., TP53) can alter the microbiome composition of LC patients, and the lung microbiome in turn can alter the expression of signaling pathways (e.g., IL-17, PI3K, MAPK, and ERK pathways) and alter the local immune microenvironment of LC tissues.

### 3.5 Gastric Cancer

Chronic HP infection leads to reduced gastric acid secretion, which may cause different bacterial communities to grow in the stomach. Is there a change in the composition of the gastric microbiota in patients with GC versus those with chronic gastritis? A study analyzing the gastric microbiota of 54 patients with GC and 81 patients with chronic gastritis found reduced microbiota diversity, reduced abundance of Helicobacter, and enrichment of gut commensal-dominated bacterial genera in GC (65). Intestinal epithelial metaplasia of the gastric is a precancerous lesion of GC, and it is worth exploring whether there is any connection between the enrichment of intestinal commensal bacteria in the gastric and intestinal epithelial metaplasia.

Chen et al. analyzed mucosa-associated microorganisms from 62 pairs of matched GC tissues and adjacent non-cancerous tissues and found increased microbial abundance and diversity in cancer tissues. The bacterial taxa enriched in cancer samples were mainly represented by oral bacteria (such as *Peptostreptococcus, Streptococcus*, and *Fusobacterium*), while lactic acid producing bacteria (e.g., *Lactococcus lactis* and *Lactobacillus brevis*) were more abundant in adjacent non-tumor tissues, and the abundance changes of oral microbiota in the stomach may be related to the development or progression of GC (66). Gunathilake et al. analyzed 268 GC cases and 288 healthy controls and found significant differences in the composition of the non-HP microbiota among the groups. Participants with higher relative abundance of *Actinobacteria* species showed a markedly increased risk of GC (67). Differences existed between GC patients and healthy controls not only in the TM, but also in the serum microbiome. The structure of the serum microbiome of GC patients was significantly different from that of patients with atypical hyperplasia, patients with chronic gastritis and healthy controls, with an enrichment of *Acinetobacter, Bacteroides, Haemophilus parainfluenzae* in GC patients, and an enrichment of *Sphingomonas, Comamonas*, and *Pseudomonas stutzeri* in healthy controls (68). The structure of the serum microbiome also showed differences in GC-Non-lymphatic Metastasis and GC-Lymphatic Metastasis (68).

The GC microbiome can promote antitumor immune responses through multiple mechanisms. Infection with HP increased PD-L1 expression in gastric epithelial cells, and exposure to HP inhibited the proliferation of CD4^+^ T cells isolated from the blood, and this inhibition could be blocked by anti-PD-L1 antibodies (69, 70). Wu et al. confirmed that HP infection enhanced PD-L1 expression in human gastric epithelial cells and that co-culture experiments of HP-infected gastric epithelial cells with primary human T cells or Jurkat T cells induced T cell apoptosis (71). HP infection may result in non-specific suppression of circulating T cells, including tumor-specific T cells (70).

### 3.6 Ovarian Cancer

Ovarian cancer (OC) is characterized by dysbiosis, in which the TM are found in many sites, including the tumor tissue itself, the upper and lower portions of the female genital tract, the serum, the peritoneum, and the intestines (72). A study analyzed the diversity and composition of the microbiota of 25 OC samples and 25 normal distal fallopian tube tissues. This study found a significant decrease in the diversity and richness indexes of OC tissues and a significant increase in the proportion of *Proteobacteria/Firmicutes phyla* in OC compared to normal distal fallopian tube tissues (73). Wang et al. tested ovarian tissues from six patients with OC and 10 patients with non-cancerous ovarian disease and confirmed the presence of bacteria in ovarian tissues by immunohistochemical staining with antibacterial lipopolysaccharide (LPS) antibody, with more Aquificae and Planctomycetes composition and reduced Crenarchaeota in OC (74). Age and BRCA1 germline mutations are known risk factors for OC, and lactobacilli species are critical for producing protective low vaginal Ph (75, 76). Nené et al. divided OC and control samples into those with at least 50% of the *lactobacilli* present (L community type) and those with less than 50% of the *lactobacilli* present (O community type). They found that the prevalence of community type O microbiota was higher in women aged 50 years or older than in those younger than 50 years, that the prevalence of community type O microbiota was significantly higher in women younger than 50 years with OC than in age-matched controls, and that in the BRCA group, women younger than 50 years with BRCA1 mutations were also more likely to have community type O microbiota than age-matched controls (76). This study suggested that having a community type O cervicovaginal microbiota was significantly associated with the patient’s age and BRCA1 germline mutation (76).

Bacteria are present in OC tissues and can produce LPS74. LPS stimulation of OC cells enhances invasion and induces production of EMT-associated cytokines, and overall survival of OC treated with LPS is even worse than that of untreated controls (77, 78). LPS is an oncogenic bacterial product in OC72. It was found that inhibition of Toll-like receptor 4 (TLR4) induced OC cell cycle arrest and apoptosis and prevented the proliferation of cancer cells (79). *L. lactis* probiotics downregulated the expression levels of TLR-4, miR-21, and miR-200b, thereby inducing apoptosis and inhibiting migration in OC cells80. Vaginal isolates of probiotic strains have great potential to control OC and may have a beneficial impact on the clinical management of OC (80). The ovarian microbiota has both a promotive and inhibitory role in the development of OC.

### 3.7 Melanoma

Mrázek et al. compared the composition of the skin microbiome in the skin and melanoma of the Melanoma-bearing Libechov Minipig model, and they found that the bacterial composition and diversity of the skin and melanoma microbiomes were significantly different, with *Fusobacterium* and *Trueperella* genera significantly more abundant in the melanoma samples (81). *Propionibacterium* genus was the most common bacterial genus in melanoma, followed by *Staphylococcus* and *Corynebacterium* (82). Another study suggested that only *Corynebacterium* was significantly associated with melanoma in patients with stage III/IV compared to those with stage I/II lesions (83). Compared with stage T1/T2 melanoma, a significant increase in *Corynebacterium* was detected in patients with stage T3/T4 melanoma, and more IL-17-positive cells were detected in *Corynebacterium*-positive patients (83). IL-17 promoted proliferation of melanoma cells through upregulation of IL-6 and signal transducer and activator of transcription (STAT) 3 (84). IL-17 induces the production of inflammatory mediators (mainly neutrophils) and stimulates the expansion and tissue infiltration of myeloid cells, thereby promoting cancer progression, and it has also been associated with the tumor microenvironment, immunotherapy, and resistance of tumor cells to radiation therapy and chemotherapy (85). The interaction of IL-17 with TM in tumor progression deserves further study in a variety of tumors. The skin microbiota of melanoma differs from that of normal skin, and *Corynebacterium* may accelerate the progression of melanoma.

### 3.8 Bone Tumors

Researchers have detected bacterial DNA in bone tumors that are not directly linked to the external environment, and analysis of the predicted MetaCyc metabolic pathway revealed that degradation of hydroxyprolines by bacteria (MetaCyc PWY-5159) was enriched in bone tumors (16). Bone collagen is a major source of hydroxyproline, and bone tumors have been shown to cause an increase in hydroxyproline levels (16, 86).

### 3.9 Brain Tumors

Bacterial DNA was present in two out of 40 glioblastoma multiforme (GBM) samples (16). Because of the blood-brain barrier, microorganisms cannot enter the brain directly, and when the gut homeostasis is perturbed, the function of the gastrointestinal tract and other organ systems (including the brain) can be impaired (87). The gut microbiota is thought to contribute to the disruption of the blood-brain barrier and the pathogenesis of neurodegenerative diseases (87). Gut microbiota metabolite alterations affect systemic and central nervous system (CNS) immunity *via* the Gut-Brain Axis (88, 89).

### 3.10 Colorectal Cancer

Compared to matched normal colon tissue samples, colorectal cancer (CRC) tissue showed increased microbial diversity in the tumor microenvironment, changes in the abundance of commensal and pathogenic bacterial taxa, including *Fusobacterium* and *Providencia*, and a significant enrichment of predicted virulence-related genes in the CRC microenvironment (90). A study analyzed CRC and matched normal tissue specimens also found a significant excess of *F. nucleatum* sequences in CRC relative to control specimens, which was positively correlated with lymph node metastasis (19). *F. nucleatum* can promote CRC metastasis through multiple pathways (including miR-1322/CCL20 axis and M2 polarization, regulation of long non-coding RNA Keratin7-antisense and Keratin7, and upregulation of caspase activation and recruitment domain 3 expression to activate autophagic signaling pathways) (91–93). *F. nucleatum* abundance correlates with high glucose metabolism in CRC patients, and *F. nucleatum* targetes lncRNA ENO1-IT1 promotes CRC glycolysis and tumor progression (94). *F. nucleatum* induced a dramatic decrease in m^6^A modification in CRC cells and patient-derived xenograft tissues through downregulation of the m^6^A methyltransferase METTL3, resulting in CRC aggressiveness (95). A study quantified F. nucleatum DNA in 181 colorectal cancer liver metastasis (CRLM) specimens and found that *F. nucleatum*-positive CRLM showed a significantly lower density of CD8^+^ T cells and a higher density of MDSCs compared to *F. nucleatum*-negative CRLM, and the difference was statistically significant, but the relationship between *F. nucleatum* and density of tumor-associated macrophages (TAMs) was not statistically significant (96). *F. nucleatum* may be a biomarker of CRC (97).

### 3.11 Papillary Thyroid Carcinoma

The presence of microbes in papillary thyroid carcinoma (PTC) tumor tissues, which are apparently lacking in adjacent normal tissues, may be critical in controlling immune cell expression and regulating immune and cancer pathways to mitigate cancer growth, and the apparent abundance of microbes in the tall cell and male patient cohorts is also associated with higher mutation expression and methylation of tumor suppressors (98).

### 3.12 Lymphomas

A study analyzed the microbiome characteristics of cutaneous T-cell lymphoma (CTCL), with no significant differences in genus level or microbial diversity compared to normal controls, and some bacterial species (*Streptomyces* sp. SM17, *Bordetella pertussis*, etc.) were determined to be more abundant in healthy-appearing skin samples (99). Another study found no significant differences in cutaneous viral or fungal communities in CTCL patients compared with age-matched healthy controls sampled at the same sites, but there were differences in changes in bacterial communities, with higher relative abundance of *Corynebacterium* spp. and lower relative abundance of *Corynebacterium* spp. in CTCL skin and high relative abundance of *C. tuberculostearicum* in stage IVA1 patients (100). Staphylococcus aureus was shown to contribute to CTCL progression (101).

The development of MALT lymphoma is closely associated with infection by microorganisms such as HP and *Chlamydophila psittaci* (CP). Antibiotic therapy against HP or CP is the first-line treatment, with lymphoma response rates of 75% to 80% after eradication of HP and 33% to 65% after antibiotic therapy for CP (102). HP infection may also be a possible cause of ocular adnexa lymphoma (OAL). Patients with OAL showed a significantly higher proportion of gastric Hp infection compared to healthy cases, suggesting that chronic local antigenic stimulation would lead to the development of ectopic B-cell lymphoma (103, 104). Tanaka et al. analyzed the microbiome of HP-negative MALT lymphoma and found that compared to controls, HP-negative MALT lymphoma patients had significantly lower alpha diversity, and *Burkholderia* and *Sphingomonas* genera were significantly more abundant in MALT lymphoma patients, while *Prevotella* and *Veillonella* genera were lower (105).

TM has been identified and studied in a variety of tumors such as PCA, LC, and BC. The microbiome is different in tumor tissues from normal tissues and influences the biological behavior of tumors by affecting the local immune system (**Table 1**), and TM may be used as a biomarker for the diagnosis and differential diagnosis of tumors in the future.

**Table 1:** Composition of the TM in tumors and its relationship to clinical features and tumor immunity.

Tumor Type	Common TM	Relationship to clinical features	Relationship with tumor immunity	References
Prostate Cancer	*Staphylococcus, Propionibacterium, Acinetobacter* and *Pseudomonas*	*Pseudomonas* infection may impede metastasis	CP1 increases T cell toxicity and immune death of tumor cells	(40,46)
Pancreatic Cancer	*Enterobacteriaceae* and *Pseudomonadaceae*	TM mediates resistance of tumor cells to gemcitabine. Patients with high TM alpha-diversity had longer overall survival.	The TM mediates anti-tumor immunity through activation of CD8^+^T cells.	(32, 38, 50)
Breast Cancer	*Pseudomonadaceae, Enterobacteriaceae, Proteus*	Lymphovascular invasion was positively correlated with *Lactobacillus* and negatively with *Alkanindiges*. In a mouse model, *Staphylococcus* and *Lactobacillus* promote lung metastasis of BC.	*Methylibium, Pelomonas, Propionibacterium* were identified as nodes in the microbiome-immune gene and microbiome-cytokine networks	(54, 55)
Lung Cancer	*Acidovorax, Veillonella parvula*,	*Acidovorax* are abundant in lung cancer patients who smoke. *Veillonella parvula* is associated with poor prognosis	*Veillonella parvula* led to the recruitment of Th17 cells, increased levels of IL-17 and PD-1^+^ T cells. Commensal bacteria induce the proliferation and activation of γδ T cells thereby promoting the proliferation of LC cells.	(18, 63, 64)
Gastric Cancer	*Peptostreptococcus, Streptococcus* and *Fusobacterium*	Changes in the abundance of oral microbiota in the stomach may be associated with the development or progression of GC.	HP infection enhanced PD-L1 expression in human gastric epithelial cells and led to non-specific suppression of circulating T cells	(66, 70)
Ovarian Cancer	*Proteobacteria, Firmicutes, Aquificae* and *Planctomycetes*	LPS stimulation of OC cells enhances invasion and induces production of EMT-associated cytokines	–	(72–74)
Melanoma	*Propionibacterium*, *Staphylococcus* and *Corynebacterium*	Compared with stage T1/T2 melanoma, a significant increase in *Corynebacterium* was detected in T3/T4 melanoma	More IL-17-positive cells were detected in *Corynebacterium*-positive patients	(83)
Colorectal cancer	*Fusobacterium* and *Providencia*	*F. nucleatum* can promote CRC metastasis through multiple pathways	*F. nucleatum*-positive CRLM showed a significantly lower density of CD8+ T cells and a higher density of MDSCs compared to *F. nucleatum*-negative CRLM	(90, 96)

## 4. TM and the treatment of tumors: future research directions

### 4.1 Effects of TM on Chemotherapy Drugs

Gemcitabine (dFdC, 2’,2’-difluorodeoxycytidine) is a deoxycytidine nucleoside analogue106. It actively crosses the cell membrane, is phosphorylated more efficiently and is eliminated more slowly, and is important in the treatment of many tumors including PC, BC, and CRC (106–109). The intracellular metabolism and anticancer activity of dFdC are affected by TM. *In vitro* experiments revealed that the efficacy of dFdC was significantly reduced in cultures of tumor cells (including BC, Murine leukemia, etc.) infected with *Mycoplasma hyorhinis* (*M. hyorhinis*) due to the rapid catabolism of the drug by cytidine deaminase (CDD) produced by *M. hyorhinis* (110). *In vivo* experiments also revealed a significant decrease in the antitumor effect of dFdC observed in BC mice whose tumors carried *M. hyorhinis* infection compared to uninfected mice (110). A study by Lehouritis et al. found that E. coli increased the effect of tegafur and decreased the effect of vidarabine, dFdC, and etoposide phosphate (111). They further conducted *in vivo* experiments with a mouse colon cancer model and showed a significant increase in tumor volume and a significant decrease in survival in the dFdC + bacteria group compared to the dFdC alone group, indicating that the antitumor activity of dFdC was reduced in tumors containing bacteria (111). Geller et al. determined that long form of CDD (CDDL) conferred microbial resistance to gemcitabine and that 12 species expressing CDDL such as EHEC, *E. coli* O157:H7, *Citrobacter freundii*, etc. were able to develop resistance to dFdC, the only bacterium that conferred resistance to dFdC despite expressing short form of CDD (CDDS) was *M. hyorhinis* (38). CDDL-deficient *E. coli* lacked the ability to metabolize dFdC, and supplementing CDDL-deficient E. coli with CDDL restored the ability to metabolize dFdC, confirming that bacteria with CDDL confer resistance to dFdC. Ciprofloxacin-treated mice (with no detectable bacteria) showed a significant antitumor response to dFdC, while control-treated mice (with detectable bacteria) showed rapid tumor progression (38). The researchers further delivered dFdC (with or without antibiotics) topically into the tumors and found significantly more apoptosis when dFdC was given in combination with antibiotics than when it was given alone. Geller et al. cultured bacteria from 15 fresh human PDAC tumors and found that 14/15 (93%) rendered RKO and HCT116 human CRC cell lines completely resistant to dFdC and that PDACs contain bacteria (*Gammaproteobacteria*) that can potentially modulate tumor sensitivity to dFdC (38, 112).

*F. nucleatum* can promote CRC metastasis through multiple pathways (91–93). *F. nucleatum* was enriched in the tissues of CRC patients who recurred after chemotherapy. *F. nucleatum* reduced CRC apoptosis induced by oxaliplatin and 5-fluorouracil and induced CRC resistance to Oxaliplatin and 5-fluorouracil (113). *F. nucleatum* may work on CRC by TLR4 and MYD (88), leading to selective loss of miR-18a* and miR-4802 expression, followed by activation of autophagy and consequently promoting chemoresistance in CRC patients (113). Phages kill oncogenic bacteria, modulate the immune system, and deliver toxins to the tumor microenvironment, and the use of phages to manipulate the TM and improve cancer treatment outcomes is a promising therapeutic measure (114).

Doxorubicin is a chemotherapy drug used in Neoadjuvant chemotherapy for BC (115). Chiba A’s study found that treatment of BC cells with *P. aeruginosa* conditioned media (P-CM), P-CM enhanced doxorubicin-mediated cell death in MDA-MB-231, 4T1 and MCF7 cell lines. Lectin (metabolite of *P. aeruginosa*), while having no significant overall effect on the proliferation of MDA-MB-231 alone, enhanced chemotherapy-mediated BC cell killing when combined with doxorubicin (115).

Some bacteria can accelerate tumor progression and cause resistance to chemotherapeutic drugs. The combination of antibiotics and chemotherapeutic drugs can effectively inhibit bacterial growth in tumors, alleviate bacterial-induced cancer resistance, and suppress tumor growth (38, 50, 91). However, the use of antibiotics not only kills the “good” bacteria in the body, which play an important role in food digestion, vitamin synthesis, and gastrointestinal motility, but also affects the role of the gut microbiota in regulating chemotherapy drugs (116–119). Zhang et al. designed a nano-reservoir loaded with both dFdC and ciprofloxacin and decorated with hyaluronic acid that can be opened in a hyaluronidase-rich tumor microenvironment (116). The nanocontainer can specifically target the tumor region to produce significant toxicity, kill intratumor bacteria and inhibit tumor growth, and exhibit good antibacterial and anticancer activities *in vitro* and *in vivo*. The nanocontainer also promoted the accumulation of active T cells in tumors and enhanced immunotherapy of tumors (116).

We already know that the gut microbiota can colonize pancreatic tumors, altering tumor bacterial composition and modulating immune function, ultimately affecting the natural course and survival of PC (32). Since the gut microbiota has a huge impact on tumors such as PC and CRC, the use of fecal microbiota transplantation therapy to alter the gut microbiota and thus TM is also a good treatment option.

The gut microbiome can influence the efficacy of a variety of drugs such as *Cyclophosphamide, Methotrexate*, and PD-1 inhibitors (13, 120). In a PC model, the deletion of TM enabled the efficacy of checkpoint-targeted immunotherapy through upregulation of PD-1 expression (50). Microbiome-centered interventions have great potential for the future of immuno-oncology (121). Whether TM can influence the efficacy of other chemotherapeutic and immunotherapeutic agents requires additional studies.

### 4.2 TM Can Be Used as Biomarkers for Cancers

TM is part of the tumor microenvironment and can influence the biological properties of the tumor through its metabolites, but can also be affected by cancer treatment (9, 16, 115, 116). A study by Kwong et al. found that bacteraemia of some microorganisms is associated with the development of CRC, and that these bacteria may enter the bloodstream from intestinal flora dysbiosis and barrier dysfunction (122). They found an increased risk of CRC in patients with the presence of *Bacteroides fragilis, Streptococcus gallolyticus, F. nucleatum*, and other bacteremia, but no increased risk in patients with bacteremia caused by microorganisms not associated with CRC (122). Using cell-free blood-based microbial DNA (mbDNA) from plasma with high discriminatory performance in healthy controls and patients with multiple types of cancer, a new class of microbial-based cancer diagnostic tools may offer substantial future value to patients (123). Studies in PTC have found specific TMs associated with higher mutation expression and methylation of tumor suppressors (98). In patients with esophageal squamous cell carcinoma (ESCC), TM with high levels of *F. nucleatum* showed a poorer response to chemotherapy, and a high *F. nucleatum* burden was associated with poor recurrence-free survival (RFS) (124). *F. nucleatum* is positively correlated with metastasis in CRC and suggests a poor prognosis (19, 125, 126). *F. nucleatum* also colonizes BC and accelerates tumor growth and metastatic progression (56). These findings suggest that the use of TM as a biomarker for cancer is promising.

### 4.3 Anticancer Effects of Engineered Tumor-Targeting Bacteria

Bacille Calmette-Guerin (BCG) is an attenuated vaccine derived from Mycobacterium bovis and is used primarily for the prevention of tuberculosis (127). The efficacy of intravenous BCG after bladder tumor resection was first reported in 1976 as superior to resection alone and resection plus intravenous chemotherapy, and the use of BCG three times a week after induction further significantly reduces tumor recurrence and patient death (128). BCG has become the gold standard for the treatment of non muscle-invasive bladder cancer (NMIBC) (129, 130). Mechanistically, BCG induces a strong innate immune response over several weeks, leading to durable anti-tumor adaptive immunity (129). This is a classic success story of the use of microbial products for oncology treatment.

Engineered tumor-targeting bacteria can specifically target tumors, actively penetrate tissue, be easily detected, and induce cytotoxicity in a controlled manner (131). Over the past few decades, Salmonella, Clostridium, and other genera have been proven to inhibit tumor growth and promote animal survival in *in vitro* experiments (132, 133). There are three main types of bacterial anticancer agents: cytotoxic agents that kill cancer cells directly, cytokines that stimulate immune cells to kill cancer cells, and tumor antigens that sensitize the immune system to cancer cells (131). Systemic administration of tumor necrosis factor alpha (TNFα) induces high levels of toxicity and causes serious side effects. Murphy et al. studied the non-pathogenic E. coli MG1655 strain as a tumor targeting system in order to specifically produce TNFα in mouse tumors. Tumor growth in three subcutaneous mouse tumor models (CT26 colon, RENCA renal, and TRAMP prostate) was impeded by injection of E. coli TNFα production constructs into mouse models *via* intratumoral or intravenous administration (134). Din et al. studied a specific phage strain that was programmed to synchronize lysis and release genetically encoded cargo when ata threshold population density was reached (135). They administered lysis strains orally to syngeneic mouse transplant models of hepatic colorectal metastases alone or in combination with clinical chemotherapeutic agents and found that the combination of circuit-engineered bacteria and chemotherapy resulted in a significant reduction in tumor activity, along with a noticeable survival advantage over either therapy alone (135). Treatment with live tumor-targeting bacteria offers a unique option for the treatment of cancer (136).

### 4.4 GEN-001

GEN-001 (*Lactobacillus lactis*) is an oral microbiota candidate therapeutic agent. Each GEN-011 capsule will contain more than 1x10^11^ colony-forming units (CFU). It is a live, purified facultative anaerobic gram-positive probiotic lactic acid bacterial strain. GEN-001 has immunomodulatory activity and it may have promising therapeutic effects against cancer through activation of immune cells, including CD4 or CD8 T cells and natural killer cells, as well as synergistic effects with oxaliplatin chemotherapy. There are currently two clinical trials related to GEN-001 that can be found on clinicaltrials.gov. One is to evaluate the efficacy and safety of total neoadjuvant therapy (TNT) in combination with GEN-001 and to investigate the dynamics of the gut microbiomes and metabolites and their effects on immune regulation (NCT05079503). The other is to evaluate the safety, tolerability, biological, and clinical activities of GEN-001 in combination with avelumab for the treatment of multiple cancer indications in a combination trial that is intended to be the first human study including both dose-escalation and expansion cohorts to assess safety and preliminary efficacy (NCT04601402). We summarized the relevant information of these two clinical trials (**Table 2**).

**Table 2:** Information about the GEN-001 clinical trial (https://clinicaltrials.gov/).

Identifier	NCT04601402	NCT05079503
Sponsor	Genome & Company	Korean Cancer Study Group
Recruitment Status	Recruiting	Not yet recruiting
Conditions	Solid Tumor, Non Small Cell Lung Cancer, Squamous Cell Carcinoma of Head and Neck, Urothelial Carcinoma	Locally Advanced Rectal Cancer
Intervention/treatment	GEN-001, Avelumab	GEN-001
Estimated Enrollment	93 participants	40 participants
Study Start Date	October 26, 2020	December 15, 2021
Estimated Study Completion Date	January 2024	January 30, 2024

**Figure 2:**
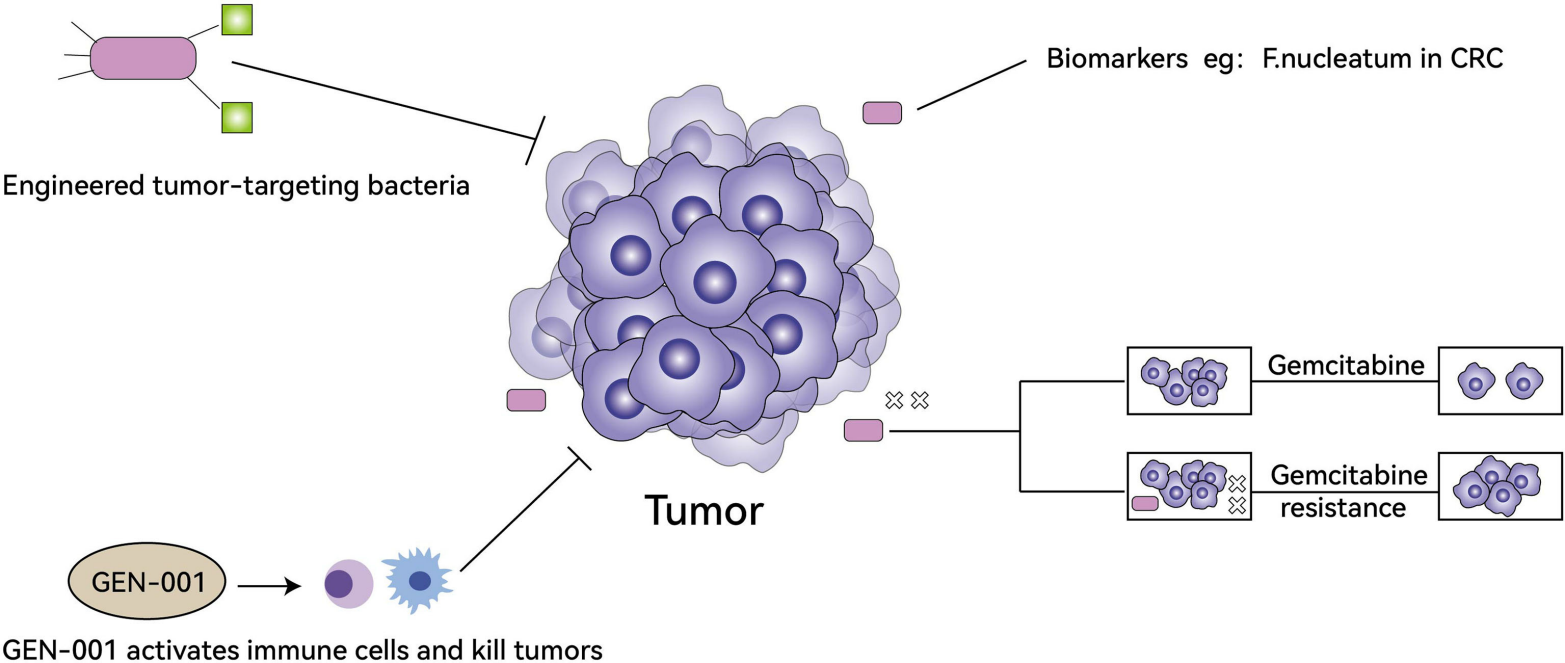
The role of TM in tumor treatment. Engineered tumor-targeting bacteria can kill tumors directly (top left), some bacteria can be used as biomarkers for treatments (top right), oral microbial antitumor agents (GEN-001) activates immune cells to synergistically kill tumors and has begun clinical trials (bottom left), TM can produce small molecules and metabolites (e.g. CDDL) to enhance tumor cell resistance to gemcitabine (bottom right).

In the treatment of tumors, TM can influence the resistance of tumor cells to drugs, engineered tumor-targeting bacteria are important research directions, some bacteria can be used as biomarkers for treatments, and oral microbial antitumor agents have started clinical trials (**Figure 2**).

## 5 Conclusion

There is a large symbiotic microbiota in humans, and with related research, it has been found that TM is also present within many tumor tissues. TM is not only closely related to the clinical features of tumors, tumor immunity, and tumorigenesis and progression, but also has great potential in the treatment of tumors. TM may promote tumor progression and may induce chemotherapy resistance, some TM (e.g., *F. nucleatum*) may become biomarkers for CRC and BC, engineered tumor-targeting bacterias are also a research direction for tumor therapy, and clinical trials of the new oral microbiota candidate GEN-001 for tumor treatment are underway. We believe that as further studies on TM are conducted, its clinical and scientific value will become more and more significant.

## Author Contributions

YC wrote and critically revised the manuscript. F-HW and P-QW drew the picture and completed the tables. H-YX and TM wrote and critically revised the manuscript. All authors contributed to the article and approved the submitted version.

## Funding

The present study was supported by grants from The Natural Science Foundation of Sichuan Province (2022NSFSC1595), The Research Foundation of The Affiliated Hospital of Southwest Medical University (15045); The Research Foundation of Southwest Medical University (2017-ZRQN-092 and 2017-ZRQN-013).

## Conflict of Interest

The authors declare that the research was conducted in the absence of any commercial or financial relationships that could be construed as a potential conflict of interest.

## Publisher’s Note

All claims expressed in this article are solely those of the authors and do not necessarily represent those of their affiliated organizations, or those of the publisher, the editors and the reviewers. Any product that may be evaluated in this article, or claim that may be made by its manufacturer, is not guaranteed or endorsed by the publisher.
